# Data Augmentation for a Virtual-Sensor-Based Nitrogen and Phosphorus Monitoring

**DOI:** 10.3390/s23031061

**Published:** 2023-01-17

**Authors:** Thulane Paepae, Pitshou N. Bokoro, Kyandoghere Kyamakya

**Affiliations:** 1 Department of Electrical and Electronic Engineering Technology, University of Johannesburg, Doornfontein 2028, South Africa; 2 Institute for Smart Systems Technologies, Transportation Informatics, Alpen-Adria Universität Klagenfurt, 9020 Klagenfurt, Austria; 3 Faculté Polytechnique, Université de Kinshasa, P.O. Box 127, Kinshasa XI, Democratic Republic of the Congo

**Keywords:** water-quality monitoring, eutrophication, synthetic data, soft sensor, surrogate variables, variational autoencoder, machine learning, deep neural network, parameter optimization

## Abstract

To better control eutrophication, reliable and accurate information on phosphorus and nitrogen loading is desired. However, the high-frequency monitoring of these variables is economically impractical. This necessitates using virtual sensing to predict them by utilizing easily measurable variables as inputs. While the predictive performance of these data-driven, virtual-sensor models depends on the use of adequate training samples (in quality and quantity), the procurement and operational cost of nitrogen and phosphorus sensors make it impractical to acquire sufficient samples. For this reason, the variational autoencoder, which is one of the most prominent methods in generative models, was utilized in the present work for generating synthetic data. The generation capacity of the model was verified using water-quality data from two tributaries of the River Thames in the United Kingdom. Compared to the current state of the art, our novel data augmentation—including proper experimental settings or hyperparameter optimization—improved the root mean squared errors by 23–63%, with the most significant improvements observed when up to three predictors were used. In comparing the predictive algorithms’ performances (in terms of the predictive accuracy and computational cost), k-nearest neighbors and extremely randomized trees were the best-performing algorithms on average.

## 1. Introduction

### 1.1. Background and Motivation

Water eutrophication, the severity of which is increasing in developing countries, has become one of the most serious water-quality problems in the world [1]. With “clean water and sanitation for all” listed among the 17 sustainable development goals, the deterioration of freshwater resources (such as rivers, lakes, and reservoirs) is of great international concern since it threatens the reliable (safe and sufficient) water supply for domestic, recreational, irrigation, or industrial uses due to the potential risk of the resulting cyanobacterial blooms on the health of end users [2,3]. While eutrophication was principally thought to result from point sources (e.g., wastewater treatment plants), recent evidence shows that diffuse phosphorous (P) and nitrogen (N) sources (e.g., urban and agricultural run-off) are key factors [4]. This is because, while point-source pollutants can be controlled at the sewage treatment plants, there has not been much progress in reducing nutrient (mainly P and N) loads from diffuse or non-point sources [4]. Therefore, from the surface-water-management perspective, reliable information on P and N loading is desired to control eutrophication.

Currently, much of the information concerning trends and patterns in nutrient loads and concentrations are based on the traditional grab-sampling approach, in which discrete water samples are manually collected from water bodies at weekly to monthly intervals before being analyzed in laboratories [5,6]. However, due to the highly dynamic nature of stream nutrients [7], the accuracy of N and P loading estimates may be inferior due to this low-frequency monitoring [8]. Therefore, even though this low-temporal-monitoring approach has yielded important water-quality information, more timely and accurate quantification of nutrient loads and concentrations could help policy-makers and resource managers to (i) identify specific pollution sources, (ii) evaluate compliance with regulatory quality objectives, (iii) develop more effective responses, and (iv) assess the progress on measures of remedial actions [5].

For this reason, modern water management requires the quick and reliable characterization of water contaminants to enable a timely response [9]. Real-time monitoring is ideal for this purpose. Apart from enabling faster response times against natural or intentional contamination warnings, real-time information can be used to identify emerging issues and pollutants, assess water-quality changes, identify trends, and achieve rapid water screening for toxic pathogens and substances. Despite the need and recent advancements in sensor technologies, the widespread adoption of real-time N and P sensors for long-term outdoor monitoring remains limited due to significant procurement and operational costs. For instance, a one-time nitrate sensor measurement costs more than USD 60,000 when considering the purchasing, operation, and data validation costs [5]. This cost is prohibitive, particularly in developing countries such as South Africa, where the national eutrophication-monitoring program monitors around 160 rivers, dams, and lakes [10]. In fact, the cost of sensors has been one of the main barriers to deploying effective monitoring networks in recent years [11].

**Figure 1:**
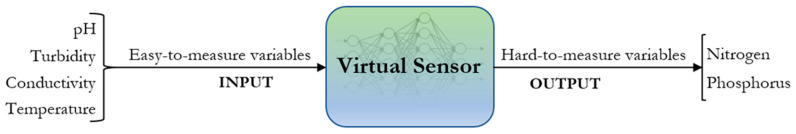
The virtual-sensing concept.

To address this central issue, easily measurable water-quality variables, such as pH, turbidity, temperature, conductivity, etc., can be employed as the inputs (also called predictors, surrogates, or auxiliary variables) of mathematical models known as virtual sensors for predicting the expensive-to-measure variables such as N and P [9]. Figure 1 illustrates the basic concept of a virtual sensor.

Although virtual sensing (i) improves the availability of the measurements, (ii) increases the measurement accuracy and reliability, and (iii) minimizes the associated measurement cost and time-related delays, its development presents several practical difficulties. One such difficulty is the poor quality and quantity of the acquired training data. For instance, the datasets that Paepae et al. [9] and Castrillo and García [12] employed when developing their nitrogen and phosphorus virtual sensors had a phosphorus-missing rate of 40% in one of the catchments, which may be the reason behind its poor prediction performance of 82% in the same catchment [9]. Further, Ha et al. [13] used 1047 observations, while Dilmi [14] had only 816 data samples to develop their nutrient (N and P) and calcium virtual sensors, respectively. This problem, which usually results in unacceptable virtual-senor performance [15], is known as a small sample problem [15,16]. With the predictive performance of data-driven virtual sensors strongly dependent on adequate training data [17,18,19], the procurement and operational cost of nitrogen and phosphorus sensors makes it impractical to acquire sufficient data for virtual-sensor modeling. In these cases, data augmentation or synthetic data generation, which generates new data samples by leveraging the existing data, may be a viable solution [19,20].

### 1.2. Literature Review

Although several preprocessing issues (such as data scaling, normalization, outlier detection, missing values handling, and input variable selection) that affect the predictive performance of virtual sensors have been studied, only a limited number of works have looked at synthetic data generation for virtual-sensor modeling. For instance, Table 1 presents the studies where extra data samples were generated for virtual-sensing purposes.

As can be seen in Table 1, deep-learning-based generative models, particularly generative adversarial networks (GANs) and variational autoencoders (VAEs), are the main methods utilized for learning generative models. Notably, the field is still emerging, as the first application of deep generative models for virtual-sensing purposes only appeared in 2019. In all the studies that compared VAEs and GANs [19,20,26], VAEs performed better. This may be attributed to the initial GAN version drawbacks, such as the training difficulty, mode collapse, gradient disappearance, and difficulty in determining when to stop training [20]. Again, As in Table 1, current applications exist in the chemical-process industry; there are none in the water-quality domain. However, due to differences in data dynamics between different problem domains, the success of a method or algorithm in one field does not guarantee the same success in another [19,27]. In this context, the suitability of VAE-based data augmentation for water-quality data remains unclear.

### 1.3. Work Objective

The primary objective of the present work is to assess the efficacy of data augmentation using a variational autoencoder on the prediction performance of N and P concentrations. To our best knowledge, this is the first study to assess the impact of a generative model on supplementing data for virtual-sensor-based nutrient monitoring. The predictive performance of the virtual sensors will then be assessed using a deep neural network (DNN), k-nearest neighbors (KNN), extreme gradient boosting (XGB), support vector regression (SVR), and extremely randomized trees (ERT) as predictive models.

The rest of the paper is organized as follows. Section 2 (i) describes the study areas, water-quality data, and data analysis frameworks, (ii) discusses the process undertaken to develop the virtual sensors, and (iii) discusses the fundamental principles of a variational autoencoder and machine-learning-based predictive models. Section 3 presents the results and discussion.

## 2. Materials and Methods

**Figure 2:**
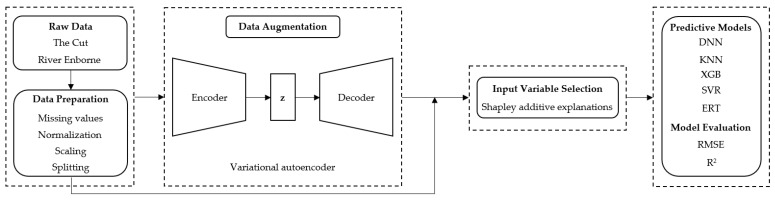
A general overview of the research framework for the present work.

Figure 2 presents a general overview of the process, from raw data acquisition to the development and evaluation of the predictive models.

A detailed description of the methods implemented in these procedures is provided in subsequent relevant sections.

### 2.1. Study Area and Water-Quality Data

The publicly available datasets utilized in the present work were obtained from [28]. The hourly physical and chemical monitoring data were measured continuously at two sites with contrasting land uses on tributaries of the River Thames: one (The Cut) draining an urban, effluent-affected system and the other (the River Enborne) draining a more rural catchment [29]. For The Cut, the data were collected from May 2010 to February 2012, while data from the River Enborne were collected from November 2009 to February 2012. Additional details, such as catchment maps, sampling locations, satellite-view photos, instrumentation characteristics, and monitoring methodology, accompany the datasets and are also provided in [29].

The variables measured in the River Enborne were pH, temperature, turbidity, conductivity, dissolved oxygen, total chlorophyll, total reactive phosphorus (TRP), and nitrogen as nitrate (NO_3_). The same variables were also measured in The Cut, except that nitrogen was measured as ammonium (NH_4_) and total phosphorus (TP) was measured only in The Cut. In both cases, datasets are accompanied by the flow-rate data taken from the closest gauging station. Regarding monitoring, TRP data in the River Enborne were measured in situ using a Systea Micromac C auto-analyser, NO_3_ was measured by UV absorption using a Hach Lange Nitratax Plus probe, and other variables were measured using a YSI 6600 multi-parameter sonde. In The Cut, TP and TRP data were also measured in situ using a Hach Lange Phosphax Sigma auto-analyser. In situ monitoring was not undertaken for NH_4_, and the remaining variables were also monitored using a YSI 6600 multi-parameter sonde [29,30]. Weekly grab sampling and laboratory analysis were undertaken for data ground truthing.

### 2.2. Data Analysis Frameworks

Due to its popularity in industrial and academic settings [31], Python (3.8.3) was used as a programming language in the present work. The following open-source software libraries, with their corresponding version numbers given in brackets, were used for data preprocessing and modeling:

1. Pandas (1.0.5): used for data manipulation and analysis;
2. TensorFlow (2.10.0): used for building a deep neural network (further discussed in Section 2.5.1);
3. Scikit-learn (1.1.0): used for implementing the k-nearest neighbors, extremely randomized trees, and support vector regression models (further discussed in Section 2.5.2, Section 2.5.3 and Section 2.5.4, respectively);
4. XGBoost (2.0.0): an implementation of a gradient-boosted decision tree algorithm;
5. PyTorch (1.12.1): used for developing the VAE. It was chosen because it has a resilient backpropagation optimizer, which was the most effective in our case.

### 2.3. Virtual Sensor Development

The available data had 20,412 records for the River Enborne and 15,636 records for The Cut. However, due to the high number of missing values (which we handled by listwise deletion, as was done by Castrillo and Garcia [12]), the resulting datasets had 12,723 and 8934 records, respectively. We then developed the virtual sensors following five main steps: (i) data preprocessing, (ii) data splitting, (iii) input variable selection, (iv) model selection, and (v) model evaluation.

#### 2.3.1. Data Preprocessing

To better fit the predictive model, a standard procedure is to preprocess the data samples first. In the present work, preprocessing included data transformation and scaling. To stabilize variance and minimize skewness in variables not normally distributed, the transformations were performed as is shown in Table 2 [9].

Additionally, all the features and targets were scaled to be between zero and one to ensure they had equal importance during training. We used the pipeline module in Scikit-learn [31] to avoid data leakage.

#### 2.3.2. Data Division

A common practice in any machine-learning experiment is to divide the available data into training, validation, and test sets. The model is trained on the training set, followed by evaluation on the validation set, and final testing on the test set when the experiment seems successful. However, dividing the data into three subsets has the drawbacks of (i) drastically reducing the number of samples available for learning the model and (ii) resulting in predictive results that depend on a specific, random choice for the training and validation sets [31]. Cross-validation solves this problem by dividing the available data into only train and test sets, where the testing set is still used for final evaluation. However, the training set is now divided into k subsets or folds, after which the model is trained using k-1 of the folds, followed by validation using the remaining set. The resulting model performance is the average computed for all the splits. We used ten-fold cross-validation and held a 20% test set in all experiments.

#### 2.3.3. Input Variable Selection

In the virtual-sensing context, selecting appropriate input variables is particularly crucial since it controls the cost of the resulting monitoring program. The chosen subset determines the predictor sensors that will be utilized for virtual sensing. Therefore, this subset is required to be optimally minimal to reduce the resulting surrogate sensors’ procurement, installation, and operational costs. We adopted the surrogates proposed in [9,12] since they are all relatively inexpensive to measure. To assess the contribution of each surrogate in the prediction of N and P concentrations, we adopted the Shapley additive explanations method for feature importance rankings, as in [9].

#### 2.3.4. Model Selection

Depending on the problem at hand, a large number of machine- and deep-learning models with varying predictive capabilities exist. An effective procedure for selecting a model is to first perform spot-checking to identify a few suitable options. In our case, a study [9] has already performed spot-checking for both catchments and identified the best-performing models. Based on their analysis, we adopted k-nearest neighbors, extremely randomized trees, support vector regression, and extreme gradient boosting models. Additionally, we included a deep neural network as a predictive model; to our best knowledge, it not been assessed for virtual-sensor-based nutrient monitoring.

#### 2.3.5. Model Evaluation

We used the coefficient of determination (R^2^) and root mean squared error (RMSE) metrics to evaluate the performance of the virtual-sensor models. They were chosen because they are the two most widely used performance metrics in water-quality research [9,31]. The formulas of the two metrics are shown in Equations (1) and (2):

(1) $$\mathrm{RMSE}\;\left(y,\hat{y}\right)\;=\;\sqrt{\frac{1}{n}\overset{n}{\underset{i\;\;=\;\;1}{\sum }}{\left(y_{i}\;-\;{\hat{y}}_{i}\right)}^{2}}$$

(2) $$R^{2}\;\left(y,\hat{y}\right)\;=\;1\;-\;\frac{\sum_{i\;=\;1}^{n}{\left(y_{i}\;-\;{\hat{y}}_{i}\right)}^{2}}{\sum_{i\;=\;1}^{n}{\left(y_{i}\;-\;\mu \right)}^{2}}$$

where $y_{i}$ denotes the actual value, ${\hat{y}}_{i}$ denotes the predicted value, n is the number of samples, and μ represents the average of the observed values.

### 2.4. Data Augmentation: A Variational Autoencoder

An autoencoder (AE) is a type of neural network designed to learn (in an unsupervised way) an identity function that can compress and regenerate original input data. It learns a more efficient data representation or latent vector by ignoring the insignificant data during compression. Contrary to traditional autoencoders that map the input data into fixed latent vector representations, VAEs map the data into the parameters of a probability distribution, such as the mean μ and standard deviation σ of a Gaussian. This produces a structured and continuous latent space useful for data generation. Therefore, while there is an architectural affinity between regular AEs and VAEs, they significantly differ in terms of the mathematical formulation [32].

#### 2.4.1. Architecture

**Figure 3:**
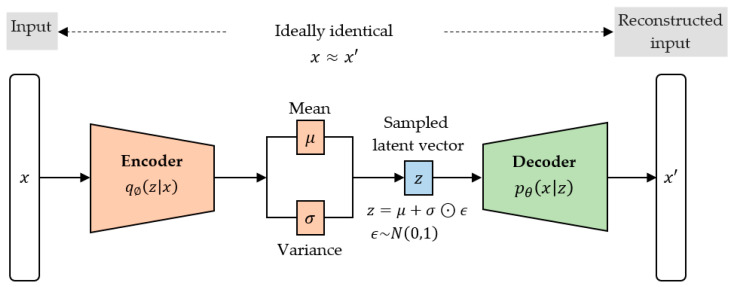
The structural framework of a variational autoencoder.

As is seen in Figure 3, the VAE consists of two main parts: an encoder and a decoder. The encoder learns to compress the input data by mapping the input x to a latent vector representation z, and the decoder learns to reconstruct the input by mapping z (sampled from the latent space) back to x’ such that x and x’ are approximately equal.

#### 2.4.2. Formulation

This section, including Section 2.4.3 and Section 2.4.4, presents the mathematical formulation of a variational autoencoder. Formally, given the input data, x, with unknown probability distribution $P\left(x\right)$, the goal is to estimate the true distribution of the P, using a distribution, $p_{\theta }$., parameterized by θ. If z is a latent vector jointly distributed with x, the relationship between the input data, *x*, and its latent representation, z, can be defined using the posterior probability, prior probability, and likelihood ratio as follows:

(3) $$p_{\theta }\left(z|x\right)\;=\;p_{\theta }\left(z\right)\;\times \;\frac{p_{\theta }\left(x|z\right)}{p_{\theta }\left(x\right)}$$

However, computing $p_{\theta }\left(x\right)$ is very expensive and intractable (i.e., requires exponential time to compute). Therefore, making the computation more feasible necessitates the introduction of a new approximation function for the posterior distribution such that:

(4) $$q_{\emptyset }\left(z|x\right)\approx p_{\theta }\left(z|x\right)$$

where $q_{\emptyset }\left(z|x\right)$ is parameterized by ∅. This way, the objective becomes finding a probabilistic autoencoder in which the conditional likelihood distribution, $p_{\theta }\left(x|z\right)$, also called a probabilistic decoder, defines a generative model while the approximated posterior distribution, $q_{\emptyset }\left(z|x\right),$ is the probabilistic encoder.

#### 2.4.3. Loss Function

Since the encoder and decoder are usually neural network models, it is crucial to utilize a differentiable loss or cost function in order to effectively update the models’ parameters (θ and ∅) through backpropagation. The objective is to jointly optimize θ (the probabilistic decoder parameters) to reduce the reconstruction error between x, x’, and ∅ to make the estimated posterior, $q_{\emptyset }\left(z|x\right),$ approximately equal to the true posterior $p_{\theta }\left(z|x\right)$. To derive the loss function, we used the reverse Kullback–Leibler divergence, (*D_KL_*), to quantify the distance between $q_{\emptyset }\left(z|x\right)$ and $p_{\theta }\left(z|x\right)$ as follows:

(5) $$D_{KL}\left(q_{\emptyset }\left(z|x\right)\;\|\;p_{\theta }\left(z|x\right)\right)\;=\;\int q_{\emptyset }\left(z|x\right)\log\frac{q_{\emptyset }\left(z|x\right)}{\;p_{\theta }\left(z|x\right)}dz$$

(6) $$\;=\;\int q_{\emptyset }\left(z|x\right)\log\frac{q_{\emptyset }\left(z|x\right)\;p_{\theta }\left(x\right)}{\;p_{\theta }\left(z,\;x\right)}dz\;\;\;\;\;\mathrm{since}\;p\left(z|x\right)\;=\;\frac{p\left(z,\;x\right)}{p\left(x\right)}$$

(7) $$\;=\;\int q_{\emptyset }\left(z|x\right)\left[\log\;p_{\theta }\left(x\right)\;+\;\log\frac{q_{\emptyset }\left(z|x\right)}{\;p_{\theta }\left(z,\;x\right)}\right]dz\;$$

(8) $$\;=\;\log\;p_{\theta }\left(x\right)\;+\;\int q_{\emptyset }\left(z|x\right)\log\frac{q_{\emptyset }\left(z|x\right)}{\;p_{\theta }\left(z,\;x\right)}dz\;\;\;\;\;\mathrm{since}\;\int q\left(z|x\right)dz\;=\;1$$

(9) $$\;=\;\log\;p_{\theta }\left(x\right)\;+\;\int q_{\emptyset }\left(z|x\right)\log\frac{q_{\emptyset }\left(z|x\right)}{p_{\theta }\left(x|z\right)p_{\theta }\left(z\right)}dz\;\;\;\;\;\mathrm{since}\;p\left(z,x\right)\;=\;p\left(x|z\right)p\left(z\right)$$

(10) $$\;=\;\log\;p_{\theta }\left(x\right)\;+\;E_{z\sim q_{\emptyset }\left(z|x\right)}\left[\log\frac{q_{\emptyset }\left(z|x\right)}{p_{\theta }\left(z\right)}\;-\;\log p_{\theta }\left(x|z\right)\right]$$

(11) $$\;=\;\log\;p_{\theta }\left(x\right)\;+\;D_{KL}\left(q_{\emptyset }\left(z|x\right)\;\|\;p_{\theta }\left(z\right)\right)\;-\;E_{z\sim q_{\emptyset }\left(z|x\right)}\left(\log p_{\theta }\left(x|z\right)\right)$$

Rearranging Equation (11) yields:

(12) $$\log\;p_{\theta }\left(x\right)\;-\;D_{KL}\left(q_{\emptyset }\left(z|x\right)\;\|\;p_{\theta }\left(z|x\right)\right)\;=\;E_{z\sim q_{\emptyset }\left(z|x\right)}\left(\log p_{\theta }\left(x|z\right)\right)\;-\;D_{KL}\left(q_{\emptyset }\left(z|x\right)\;\|\;p_{\theta }\left(z\right)\right)$$

The lefthand side of Equation (12) is what we aim to optimize when learning the true distributions. That is, we want to simultaneously maximize the log-likelihood of generating real data while minimizing the divergence between the approximate and true posterior distributions. Negating Equation (12) defines the loss or cost function as follows:

(13) $$\begin{array}{cc} L_{VAE}\left(\theta ,\emptyset \right) & \;=\;-\log\;p_{\theta }\left(x\right)\;+\;D_{KL}\left(q_{\emptyset }\left(z|x\right)\;\|\;p_{\theta }\left(z|x\right)\right)\\ & \;=\;\;-\;E_{z\sim q_{\emptyset }\left(z|x\right)}\left(\log p_{\theta }\left(x|z\right)\right)\;+\;D_{KL}\left(q_{\emptyset }\left(z|x\right)\;\|\;p_{\theta }\left(z\right)\right) \end{array}$$

The first term denotes the reconstruction likelihood, while the second term ensures that the distributions $q_{\emptyset }\left(z|x\right)$ and $p_{\theta }\left(z|x\right)$ are similar.

#### 2.4.4. Reparameterization Trick

As is seen in Figure 3, z is sampled from $q_{\emptyset }\left(z|x\right)$. However, this sampling operation is stochastic and therefore non-differentiable. Consequently, the model gradients cannot be backpropagated. To solve this problem, Kingma and Welling [33] proposed a reparameterization trick or technique. Firstly, it ensures that $q_{\emptyset }\left(z|x\right)$ is chosen to be a continuous and differentiable multivariate Gaussian so that the epsilon, ϵ, can be sampled from a standard normal distribution $N\left(0,1\right)$. Secondly, z is then generated as z = μ + σ⊙ϵ, where μ, σ and ϵ enable the model gradients to be backpropagated through μ and σ while simultaneously maintaining the stochasticity through ϵ.

#### 2.4.5. Implementation

Several parameters need to be optimized for neural-network-based encoder and decoder networks to function effectively. Following some existing works [34], we optimized the number of hidden layers, number of neurons per hidden layer, batch size, number of training epochs, optimization algorithm, learning rate, activation function, regularization, and weight initialization technique. The optimal settings are provided in Section 3.1.2. We refer the reader to [35] should they be unfamiliar with these parameters.

### 2.5. Predictive Models

Following the data augmentation step, the training and extensive testing of candidate virtual sensor models (also called predictive models) is required. Herby, we implemented several predictive models: deep neural network (a multilayer perceptron architecture), k-nearest neighbors, extremely randomized trees, support vector regression, and extreme gradient boosting.

#### 2.5.1. Deep Neural Network (DNN)

**Figure 4:**
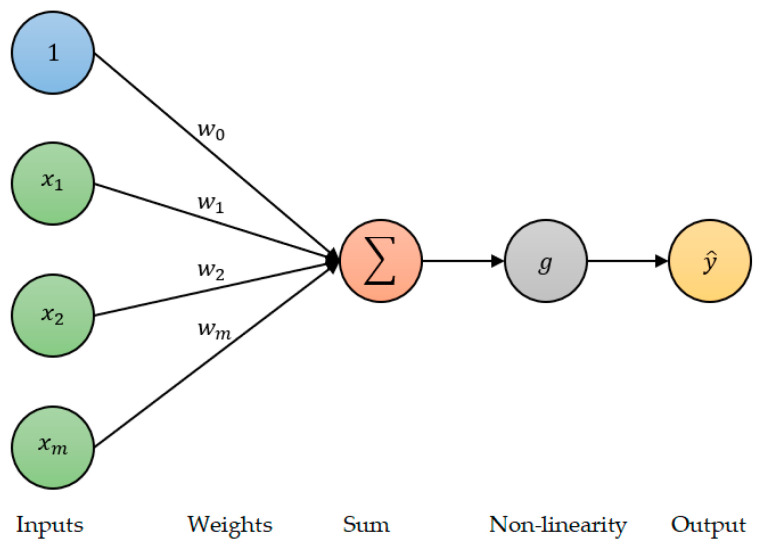
A single neuron.

A neuron (or perceptron), which is shown in Figure 4, is the fundamental building block of a deep neural network (DNN).

**Figure 5:**
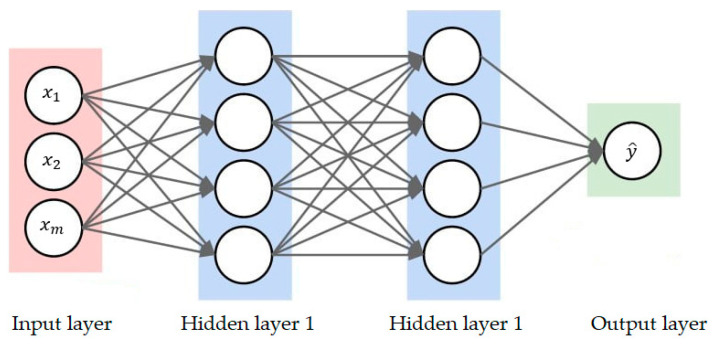
The architecture of a DNN.

To predict the output $\hat{y}$, the perceptron takes the sum of the inputs ($x_{1}\;\;\mathrm{to}\;\;x_{m})$ multiplied by their corresponding weights ($w_{1}\;\mathrm{to}\;w_{m})$, adds a bias $w_{0}$, and then passes this sum through a non-linear activation function g. Mathematically, this is presented as:

(14) $$\hat{y}\;=\;g\left(w_{0}\;+\;\overset{m}{\underset{i\;=\;1}{\sum }}w_{i}x_{i}\right)$$

(15) $$\;\;\;\;\;\;\;\;\;\;\;\;=\;g\left(w_{0}\;+\;W^{T}X\right)$$

where $W^{T}$ represents a row vector of the weights and X represents a column vector of the input features. A DNN is then created by stacking connected layers of these perceptrons with multiple neurons per layer, as is seen in Figure 5.

As is seen in Figure 5, the final architecture (excluding the bias for simplicity) contains the input layer (for bringing input features into the network for further processing), the hidden layers (for computing the weights and biases), and the output layer (for producing the predictions). In the forward pass, the predicted value $\hat{y}$ is compared with the true value y and the loss function, which is written in terms of the mean squared error, is then optimized. This optimization process, which aims to find the network parameters (weights and biases) that lead to the lowest loss, happens through a gradient descent algorithm. The parameter gradients are then updated through backpropagation in the backward pass. Similar to VAEs, several parameters must be optimized for DNNs to work effectively. Following existing works [34,36], we therefore optimized the same parameters as in the VAE, except for the regularization and learning rate (which were excluded in the DNN model. The results are provided in Section 3.1.1.

#### 2.5.2. K-Nearest Neighbors (KNN)

Contrary to DNNs, there is no learning required in the KNN algorithm since it has no model. Instead, the principle behind the KNN algorithm is that predictions for a new instance are made using the entire training set by averaging the distance from a new instance and k closest points (the nearest neighbors) [31]. We optimized (i) the number of neighbors to use, (ii) the metric to use for computing the distance, and (iii) whether the contribution of each neighboring point should be uniform or based on distance. The results are discussed in Section 3.1.3.

#### 2.5.3. Extremely Randomized Trees (ERT)

An ETR regressor is an ensemble algorithm that independently builds several decision-tree-based estimators and then averages their resulting predictions [31]. It works similarly to the commonly applied random forest (RF) [12,13,37], but has two subtle differences. For instance, an RF constructs several decision trees over bootstrapped subsets of the training data, while an ERT constructs several trees over the entire dataset. Additionally, an RF considers the best split when splitting the nodes, while an ERT randomizes the splits. We optimized the number of trees in the forest and the number of features that would result in the best split. The results of this optimization are discussed in Section 3.1.4.

#### 2.5.4. Support Vector Regression (SVR)

Support vector machines (SVMs) are established methods in supervised machine learning. Although SMVs were initially developed to solve classification problems, a formulation for regression problems exists: it is called SVR. The goal of SVR is to find a hyperplane that holds maximum training data within the margin ε [31]. The created hyperplane is situated in the middle of an ε-insensitive tube, in which the training samples outside this tube—which are the only ones considered when computing the error—are called slack variables ($\xi_{i}\;\mathrm{and}\;\xi_{i}^{*}$). To develop the most robust SVR models, the slack variables and a kernel ϕ are included in the formulation of the cost function as [14,38]:

(16) $$\underset{\left(w,b,\xi_{i},\xi_{i}^{*}\right)}{\min}\;\frac{1}{2}\|w^{2}\|\;+\;C\overset{n}{\underset{i\;=\;1}{\sum }}\left(\xi_{i}\;+\;\xi_{i}^{*}\right)$$

subject to:

(17) $$\left\{\begin{array}{c} y_{i}\;-\;\left(w^{T}\phi \left(x_{i}\right)\;+\;b_{i}\right)\;\leq \;\varepsilon \;+\;\xi_{i}\\ \left(w^{T}\phi \left(x_{i}\right)\;+\;b_{i}\right)\;-\;y_{i}\;\leq \;\varepsilon \;+\;\xi_{i}^{*}\\ \xi_{i},\xi_{i}^{*}\;\;\;\;\;\;\;\;\;\;\;\;\;\;\;\;\;\;\;\;\;\geq \;0 \end{array}\right.$$

where the constant C, which must be strictly positive, acts as a regularization parameter. Notably, samples whose $\hat{y}$ is at least ε away from y penalize the cost by $\xi_{i}$ or $\xi_{i}^{*}$, depending on whether $\hat{y}$ lies above or below the ε-sensitive tube [31]. We optimized the kernel, C, and gamma. The results are discussed in Section 3.1.5.

#### 2.5.5. Extreme Gradient Boosting (XGB)

An XGB is an ensemble learning method that predicts $\hat{y}$ by combining the predictions of regression-tree-based weaker learners built in series [39]. It minimizes a regularized cost function based on the difference between y and $\hat{y}$. The iterative training proceeds so that each subsequent sequential tree reduces the errors or residuals of the prior tree. That is, the next tree learns from the updated version of the residuals. It uses gradient descent to minimize the loss, hence the name “gradient boosting.” We optimized only the booster parameters, and the results are discussed in Section 3.1.6.

## 3. Results and Discussion

### 3.1. Model Optimization

In machine learning, a choice of model parameters can significantly influence the prediction performance. While parameters such as weights and biases are learned during model training, other parameters that control the learning process must be chosen a priori. A process of selecting an optimal set of these latter parameters, which is sometimes overlooked in water-quality research [36], is known as hyperparameter tuning or optimization. Although various approaches exist for this tuning process, we adopted the common grid and random-search approaches in the present work. We selected two because no one approach is consistently superior to others [40].

#### 3.1.1. DNN Optimization

Hyperparameter tuning is a crucial and notably difficult part of training deep neural networks [36]. This is because these networks have several parameters that need to be configured and are therefore computationally very expensive. As has already been mentioned, we optimized the network structure (the number of hidden layers and neurons per layer), batch size, number of epochs, optimization algorithm, activation function, and weight initialization technique. Table 3 presents the values of the resulting search process.

**Figure 6:**
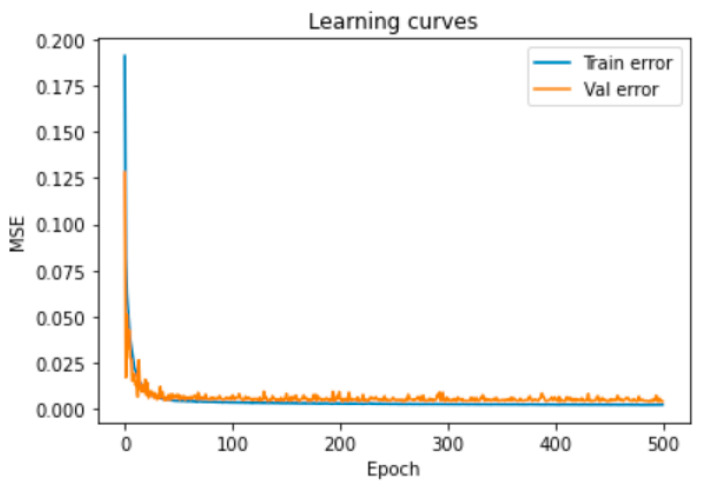
DNN learning curves for the River Enborne.

To ensure that the model was neither under- nor over-fitting, which are two prominent issues in training DNNs [41], we checked the training and validation losses, shown in Figure 6, in which MSE denotes the mean squared error. As can be seen in Figure 6, the developed neural network generalized well to the testing data without any regularization.

#### 3.1.2. VAE Optimization

Similar to DNNs, VAEs are computationally expensive to train due to a high number of hyperparameters. However, unlike in DNNs, for which we minimized the MSE, the loss function given in Equation (13) was minimized in this case. We also included batch normalization and the learning rate at this time. Table 4 presents the values of the resulting hyperparameter search process.

A behavior similar to what can be seen in Figure 6 was also observed. Interestingly, the batch size dropped significantly on the VAE. This is because TensorFlow, which was used for developing the DNN, is the most efficient when operating on large batches of data [42]. The number of epochs also dropped in the VAE. This occurred due to the computational cost involved in training the VAE models, particularly at this small batch size. In any case, the training and validation losses decreased only marginally from the 120th epoch.

#### 3.1.3. KNN Optimization

The number of neighbors to use (k), the weight function used in prediction, and the metric for computing the distance were the three hyperparameters we optimized in this work. The weight function can either be uniform or distance-based, in which uniformity implies that all the neighboring points are weighted equally, while distance weights the points based on how far they are from a query point, with closer points having a more significant influence than those further away [31]. Table 5 compares the performance obtained with default values in Scikit-learn with the performance obtained through hyperparameter tuning.

As can be seen in Table 5, hyperparameter optimization improved the RMSE by 20%. Considering that both datasets had many outliers [9], it makes sense that the optimal weight function would be distance and not the default (uniform-based). Compared to the default Minkowski-based metric, it also makes sense that the algorithm performed better with the Manhattan-based distance metric as its computation is not affected by squares and square root operations, which emphasize the impact of the already high errors due to the presence of outliers. Apart from the improved RMSE, this tuning process also proved why using only one optimization technique is sometimes insufficient. For instance, the most widely used grid search method proposed the optimal number of nearest neighbors as nine (instead of three), which leads to a comparatively worse RMSE of 0.0175.

#### 3.1.4. ERT Optimization

The two crucial parameters to optimize when using the ERT method are the number of trees in the forest (n_estimators) and the number of features that would result in the best split (max_features) [31]. Table 6 compares the predictive performance obtained with default settings in Scikit-learn and those obtained by hyperparameter tuning.

As can be seen in Table 6, the max_features remained auto after hyperparameter tuning, while n_estimators increased to 700. Contrary to the KNN algorithm, for which the resulting performance improved significantly, this increase in n_estimators improved the performance marginally (i.e., a 2% improvement in RMSE), with the downside of an increased computational cost. It is also interesting to note that the performance remained constant from when the number of trees was 200 until the number of trees reached 700. This suggests that it is ideal to further test the values on either side of the proposed optimal from the searched parameters.

#### 3.1.5. SVR Optimization

The kernel, gamma, and C are typical hyperparameters to consider when optimizing SVMs [31]. The kernel introduces flexibility; gamma is the kernel coefficient, and C is a regularization parameter with a regularization strength inversely proportional to C [31]. Table 7 compares the predictive performance obtained with default settings in Scikit-learn and those obtained by hyperparameter tuning.

As in Table 7, the gamma and kernel remained the same after hyperparameter tuning, while C increased to 200. This increase improved the RMSE by 7%. Understandably, the kernel and gamma remained the same after optimization as the radial basis function is generally the most robust kernel [31], while the inclusion of variance in the computation of the scale-based gamma may be the reason behind its increased flexibility when compared to the auto-based gamma, which does not factor the variance of features. Additionally, as a regularization parameter, lower values of C create simpler decision functions by encouraging larger margins, albeit at the expense of training accuracies [31]. Therefore, it makes sense that the model performed better at a larger value of C.

#### 3.1.6. XGB Optimization

Based on the official documentation, running an XGB model requires setting three types of parameters: general, booster, and task parameters [39]. General parameters relate to the choice of a booster, booster parameters depend on the chosen booster, and learning task parameters determine the learning scenario. In this work, we only optimized the booster parameters—max depth, n_estimators, and learning rate—since they alone significantly improved the predictive performance, as can be seen in Table 8.

As can be seen in Table 8, decreasing the learning rate while increasing the maximum tree depth and n_estimators improved the RMSE by 24%. This phenomenon is understandable since increasing the max depth and n_estimators makes the model more complex, while the step-size shrinkage brought by the learning rate prevents overfitting [39]. Overall, the optimization results discussed in this section proved the importance of hyperparameter tuning, which is usually overlooked in related nutrient-predictive studies [9,37].

### 3.2. Likeness between Real and Generated Samples

**Figure 7:**
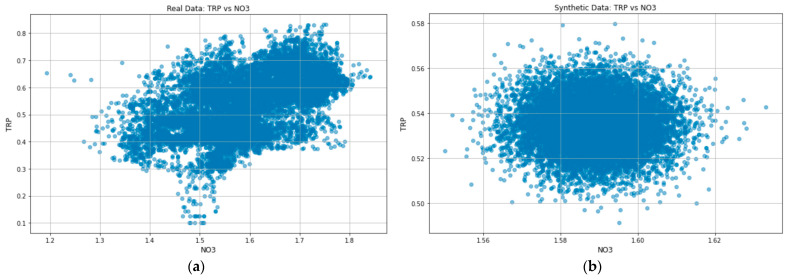
(**a**) The original data-distribution plot for TRP versus NO_3_ in the River Enborne and (**b**) the synthetic data distribution plot for TRP versus NO_3_ in the River Enborne.

Generating synthetic data according to the distribution of the underlying data is crucial since the uniformity of the generated samples is a significant factor in high-quality samples [22]. To guarantee this uniformity, it becomes essential to consider the likeness of real and generated samples. With no clear metric for an appropriate measure for this likeness [19], we used the distribution plots to check that the generated synthetic data is sampled from the original data distribution, as is shown in Figure 7.

**Figure 8:**
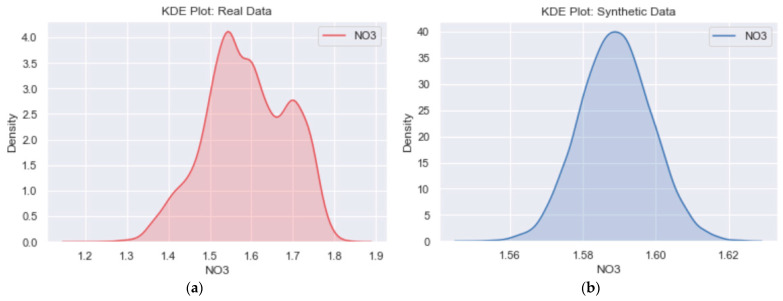
(**a**) The NO_3_ KDE plot for original data and (**b**) the NO_3_ KDE plot for synthetic data.

Although the distribution shape of Figure 7b looks different from Figure 7a, it is essential to note that the encoder model compresses the high-dimensional input data into a lower dimension. Including the Kullback–Leibler divergence term in the loss function ensures that the learned means and standard deviations are almost identical to those of the normal distribution. Doing so forces the model to express the training data more compactly and group similar data around the center of the latent space, creating a continuous space from which to sample [43]. Thus, comparing the data axes in Figure 7b and Figure 7a, it is evident that the synthetic data is indeed sampled from the original data distribution. When compared to Figure 8a, which shows the NO_3_ kernel distribution estimation (KDE) plot for real data, the KDE plot in Figure 8b further confirms that the learned means and standard deviations are close to those of the normal distribution.

Comparing Figure 8b to Figure 8a, the x-axis range of Figure 8b lies within that of Figure 8a, with a bell-shaped curve confirming its normality distribution. Although we only used total reactive phosphorus and nitrate to discuss the likeness between the original and synthetic data, the distribution plots for other variables in both catchments also agree with the discussed observations.

To assess how combining synthetic and original data affects the variable distributions, we computed the Jensen–Shannon divergence (JSD) to measure the probability distribution similarities between the original datasets and the datasets progressively augmented until we reached a doubling of the original dataset sizes. Table 9 shows the JSD between the original and progressively augmented datasets for each variable in both catchments. We excluded pH data from The Cut and chlorophyll data from both catchments as they both have very low JSD values.

As can be seen in Table 9, the progressive increase in dataset sizes marginally drops the JSD values in both catchments, with the exception of the DO and pH values in the Enborne, whose values remain constant. Although insignificant, the marginal drop in the similarity between the two probability distributions may be attributed to the decrease in data variability inherent to the VAE as the JSD is based on the Kullback–Leibler divergence [44].

### 3.3. Virtual Sensor Performance with Increasing Dataset Size

To assess the efficacy of the VAE-based data augmentation on the prediction performance of N and P concentrations, we (i) analyzed the RMSE changes in predictive models as the size of the datasets increased gradually until the sizes were doubled, and (ii) computed the percentage improvement by comparing the predictive performances at the original sizes (8934 samples for The Cut and 12,723 for the River Enborne) and doubled sizes. The comparative results are shown in Table 10.

As can be seen, the RMSE improvements after data augmentation, which monotonously increased in direct proportion to increasing datasets size, range from 22% to 31% in The Cut and 10% to 35% in the River Enborne. On average, the improvements to The Cut are understandably high compared to those of the River Enborne. This is because The Cut has a relatively small dataset compared to the River Enborne, and therefore increasing its size creates sufficient training information for the predictive models to learn from [36]. Notably, the biggest improvement in all the models was observed in the DNNs. This is also understandable since they, by design, require a large number of samples to effectively find optimal hyperparameters in complex datasets [21]. In comparing the predictive performances of the individual algorithms, the KNN algorithm performed better in The Cut, while the ERT model performed better in the River Enborne. This makes sense, as augmenting the smaller-sized datasets via the VAE supports the distance-based KNN, which groups similar data around the center of the latent space.

On the other hand, it is worth noting that our developed DNN is not the most robust since we constrained the network structure and number of epochs due to the inherent computational cost. That is, increasing the network structure and the number of epochs gradually increased the predictive performance. However, this came at the expense of very long training times, resulting in the search space being constrained to the values provided in Table 3. Thus, although DNNs may eventually outperform KNN and ERT, the high cost of computing makes them less favorable since the performance of virtual sensors is not only a function of inexpensive surrogate sensors, reliability, and acceptable accuracy, but also depends on the complexity of the developed predictive model [6,9,12].

### 3.4. Performance Based on Predictor Importance: Comparison with the Current Benchmark

Since the procurement and operational cost of virtual sensors depends on the factors mentioned, we used the best-performing algorithms (KNN for The Cut and ERT for River Enborne) to assess the impact of each surrogate in predicting N and P concentrations. As was mentioned in Section 2.3.3, we adopted the Shapley additive explanations method for feature importance rankings, as was the approach used in [9]. To further highlight the impact of the VAE-based data augmentation, we also compared the performance of the current state of the art [9] with that obtained with the doubled dataset sizes, shown in Table 11.

From Table 11, it can be seen that, when compared to the benchmark, the RMSEs in both catchments improved significantly. These improvements are notable since the predictive accuracy in urban catchments such as The Cut is usually inferior [9,12]. Interestingly, the most significant improvements were observed when up to three predictors were used. Bearing in mind that funding has not necessarily increased amid the need or calls for accurate and inexpensive monitoring systems [11], using only three surrogates at increased prediction accuracies is encouraging for water-resource managers, as only a minimal subset of surrogate sensors would provide the desired level of monitoring accuracy.

Contrary to RMSEs, which decreased gradually as the number of predictors increased, the decrease in R^2^ values was very rapid. This contrasting behavior is due to the way that VAEs generate synthetic data. Generally, the chemistry of flowing rivers is highly dynamic and exhibits periodic or seasonal variability [37]. By grouping similar data around the center of the latent space, the VAE ignores this variability; this is reflected by the rapid drop in R^2^ since its computation, as can be seen in Equation (2), represents the proportion of the *y* variance explained by the features in the model [31]. It measures how well the model will likely predict the unseen samples through the proportion of explained variance.

## 4. Conclusions

The present work used the prominent VAE model to supplement virtual-sensing data. The significant improvement in the predictive results (when compared to the baseline) and a marginal drop in the probability distribution between the real and generated data proved the effectiveness of the model. This work also demonstrated the importance of a procedure (parameter tuning) usually overlooked in N- and P-predictive studies. Based on the results, proper experimental settings improved the performances by as much as 24%. Furthermore, the performance improvement for all the algorithms monotonously increased as the datasets were augmented progressively until a doubling of the original datasets was reached.

Generally, the KNN algorithm performed better in The Cut, while the ERT algorithm was the most accurate model in the River Enborne. However, while the common belief in planning and management is that the most appropriate methods are usually simpler, the most complex models may be the only solution in urban catchments. For instance, the highest R^2^ value for TP and TRP of The Cut is only 86%. Therefore, utilizing an optimally trained DNN may improve this performance, albeit at a higher computational cost.

Although the impact of the developed VAE model is positive, when considering limitations it must be noted that there is still scope for some improvement, particularly in The Cut. For instance, contrary to relatively lower training and validation losses in the Enborne, the loss functions in The Cut did not optimally converge (the minimum training and validation losses were 1.013 and 1.007, respectively).

Additionally, considering how dynamic stream nutrients are, the VAE-generated data may not be representative, especially when the sample sizes are very small. This is because VAEs cluster data at the center of the latent space, thereby undermining data variability. This variability is important since water-quality data varies at interannual time scales. Therefore, future works will assess the efficacy of (i) an ensemble of a VAE and a GAN as a generative model and (ii) an optimally trained DNN, including deep belief networks and deep echo state networks as predictive models, especially for The Cut.

## Figures and Tables

**Table 1:** A list of studies that augmented data for virtual-sensing purposes.

Ref.	Year	Generative Models	Application
[21]	2019	Stacked autoencoder	Hydrocracking process
[19]	2020	Metropolis–Hastings algorithm,	Thermal power-plant boiler
		variational autoencoder (VAE),	
		generative adversarial network (GAN), VAE-GAN	
[20]	2021	GAN, stacked VAE (SVAE), SVAE-GAN	Thermal power-plant boiler
[22]	2021	Centroidal Voronoi tessellation sampling,	Polyethylene process
		conditional GAN (CGAN)	
[23]	2021	CGAN	High-density polyethylene
[24]	2021	Monte Carlo with particle swarm optimization,	Purified terephthalic acid
		noise-injection, target-relevant AE, VAE	Ethylene production system
[25]	2022	Combined AE data augmentation strategy	Industrial debutanizer
[26]	2022	VAE, GAN	Industrial reformer

**Table 2:** Variable transformation in each catchment.

Variable	Transformation	
	The Cut	River Enborne
Turbidity	Reciprocal	Reciprocal
Flow rate	Reciprocal	Logarithm
Chlorophyll	Logarithm	Logarithm
Dissolved oxygen	Square root	Logarithm
Nitrate (as NH_4_ or NO_3_)	Cube root	Cube root
Total Reactive Phosphorus	None	Cube root
pH	None	Reciprocal
Temperature	None	None
Conductivity	None	None
Total Phosphorus	Square root	

**Table 3:** Optimal hyperparameters of our deep neural network.

Hyperparameter	Value
Hidden layers	4
Hidden neurons	50,75,100,200
Activation function	Rectified linear unit
Batch size	300
Number of epochs	500
Weight initialization	Normal
Optimization algorithm	Root mean square propagation

**Table 4:** Optimal hyperparameters of a variational autoencoder.

Hyperparameter	Value
Encoder and decoder hidden layers	3
Encoder and decoder neurons	50,15,12
Activation function	Rectified linear unit
Latent dimensions	2
Learning rate	0.01
Batch size	4
Number of epochs	200
Weight initialization	Normal
Optimization algorithm	Resilient backpropagation

**Table 5:** NO_3_ prediction performance obtained through the KNN default and the optimized hyperparameter settings for the River Enborne, using the original data.

Default Settings		Performance		Optimized Settings		Performance	
Parameter	Value	RMSE	R^2^	Parameter	Value	RMSE	R^2^
k	5	0.0183	0.9656	k	3	0.0146	0.9781
Weight	Uniform			Weight	Distance		
Metric	Minkowski			Metric	Manhattan		

**Table 6:** NO_3_ prediction performance obtained through the ERT default and the optimized hyperparameter settings for the River Enborne, using the original data.

Default Settings		Performance		Optimized Settings		Performance	
Parameter	Value	RMSE	R^2^	Parameter	Value	RMSE	R^2^
n_estimators	100	0.0142	0.9796	n_estimators	700	0.0139	0.9802
max_features	Auto			max_features	auto		

**Table 7:** NO_3_ prediction performance obtained by through SVR default and the optimized hyperparameter settings for the River Enborne using the original data, in which rbf is the radial basis function.

Default Settings		Performance		Optimized Settings		Performance	
Parameter	Value	RMSE	R^2^	Parameter	Value	RMSE	R^2^
Kernel	rbf	0.0369	0.8611	Kernel	rbf	0.0342	0.8806
Gamma	Scale			Gamma	Scale		
C	1			C	200		

**Table 8:** NO_3_ prediction performance obtained by the XGB default and the optimized hyperparameter settings in the River Enborne using the original data.

Default Settings		Performance		Optimized Settings		Performance	
Parameter	Value	RMSE	R^2^	Parameter	Value	RMSE	R^2^
Max depth	6	0.0209	0.9554	Max depth	10	0.0159	0.9740
n_estimators	100			n_estimators	900		
Learning rate	0.3			Learning rate	0.05		

**Table 9:** The Jensen–Shannon divergence between the original and progressively augmented datasets for each variable in both catchments.

**Variable**	**The Cut**				
	**Original Size 8934**	**Increased by 2234**	**Increased by 4468**	**Increased by 6702**	**Increased by 8934**
TRP	0.0344	0.0312	0.0288	0.0274	0.0260
TP	0.0073	0.0066	0.0061	0.0058	0.0055
EC	0.0028	0.0026	0.0024	0.0023	0.0022
Turb	0.1436	0.1306	0.1226	0.1161	0.1105
DO	0.0044	0.0040	0.0037	0.0035	0.0034
Temp	0.0248	0.0224	0.0207	0.0196	0.0187
NH_4_	0.0073	0.0067	0.0061	0.0057	0.0055
**Variable**	**River Enborne**				
	**Original Size 12,723**	**Increased by 3181**	**Increased by 6362**	**Increased by 9543**	**Increased by 12,723**
TRP	0.0108	0.0098	0.0090	0.0085	0.0081
EC	0.0069	0.0062	0.0058	0.0055	0.0052
Turb	0.0602	0.0545	0.0507	0.0476	0.0457
DO	0.0003	0.0003	0.0003	0.0003	0.0003
pH	0.0002	0.0002	0.0002	0.0002	0.0002
Temp	0.0501	0.0451	0.0420	0.0395	0.0379
NO_3_	0.0010	0.0009	0.0008	0.0008	0.0007

**Table 10:** Performance comparison (in terms of the RMSE) of various virtual-sensor models with increasing sizes of synthetic data.

**Model**	**NH_4_ in The Cut**					**Predictive Performance Improvement**
	**8934**	**Increased by 2234**	**Increased by 4468**	**Increased by 6702**	**Increased by 8934**	
SVR	0.0704	0.0671	0.0619	0.0584	0.0550	22%
KNN	0.0337	0.0308	0.0290	0.0274	0.0260	23%
XGB	0.0426	0.0409	0.0387	0.0356	0.0332	22%
ERT	0.0379	0.0349	0.0326	0.0306	0.0288	24%
DNN	0.0439	0.0383	0.0332	0.0308	0.0302	31%
	**TRP in The Cut**					
SVR	0.1169	0.1074	0.1005	0.0945	0.0895	23%
KNN	0.0781	0.0708	0.0663	0.0612	0.0582	25%
XGB	0.0829	0.0751	0.0690	0.0653	0.0611	26%
ERT	0.0790	0.0713	0.0661	0.0616	0.0583	26%
DNN	0.0905	0.0818	0.0766	0.0682	0.0622	31%
	**TP in The Cut**					
SVR	0.0710	0.0645	0.0601	0.0563	0.0532	25%
KNN	0.0477	0.0432	0.0404	0.0374	0.0355	26%
XGB	0.0516	0.0460	0.0425	0.0401	0.0380	26%
ERT	0.0489	0.0440	0.0407	0.0380	0.0359	27%
DNN	0.0585	0.0532	0.0494	0.0424	0.0407	30%
**Model**	**NO_3_ in the Enborne**					**Predictive Performance Improvement**
	**12,723**	**Increased by 3181**	**Increased by 6362**	**Increased by 9543**	**Increased by 12,723**	
SVR	0.0342	0.0317	0.0295	0.0281	0.0270	21%
KNN	0.0146	0.0141	0.0136	0.0133	0.0132	10%
XGB	0.0166	0.0161	0.0152	0.0147	0.0142	14%
ERT	0.0140	0.0134	0.0128	0.0125	0.0121	14%
DNN	0.0365	0.0297	0.0268	0.0256	0.0236	35%
	**TRP in the Enborne**					
SVR	0.0395	0.0368	0.0346	0.0332	0.0322	18%
KNN	0.0215	0.0201	0.0191	0.0183	0.0177	18%
XGB	0.0224	0.0208	0.0195	0.0183	0.0175	22%
ERT	0.0203	0.0188	0.0177	0.0169	0.0163	20%
DNN	0.0318	0.0267	0.0264	0.0228	0.0223	30%

**Table 11:** Comparative performance of the present work and benchmark [9] based on the contribution of each surrogate in predicting P and N concentrations.

Predictors	Benchmark		This Work		Improvements of This Work Compared to a Benchmark	
	RMSE	R^2^	RMSE	R^2^	RMSE	R^2^
**NH_4_ in The Cut**						
Temp	0.1312	0.1620	0.0882	0.1022	33%	−59%
Temp, Chl	0.1342	0.1220	0.0681	0.4634	49%	74%
Temp, Chl, Turb	0.0907	0.5986	0.0493	0.7190	46%	17%
Temp, Chl, Turb, EC	0.0655	0.7895	0.0376	0.8362	43%	6%
Temp, Chl, Turb, EC, DO	0.0526	0.8647	0.0310	0.8887	41%	3%
Temp, Chl, Turb, EC, DO, pH	0.0429	0.9101	0.0260	0.9216	39%	1%
**TP in The Cut**						
EC	0.1213	0.1697	0.0924	0.0513	24%	−231%
EC, DO	0.1291	0.0593	0.0737	0.3961	43%	85%
EC, DO, Turb	0.0956	0.4853	0.0585	0.6196	39%	22%
EC, DO, Turb, Temp	0.0680	0.7382	0.0434	0.7900	36%	7%
EC, DO, Turb, Temp, Chl	0.0610	0.7880	0.0390	0.8308	36%	5%
EC, DO, Turb, Temp, Chl, pH	0.0556	0.8253	0.0355	0.8593	36%	4%
**TRP in The Cut**						
EC	0.1952	0.1820	0.1506	0.0399	23%	−356%
EC, Turb	0.2037	0.1072	0.1127	0.4609	45%	77%
EC, Turb, DO	0.1554	0.4813	0.0955	0.6136	39%	22%
EC, Turb, DO, Temp	0.1101	0.7401	0.0704	0.7897	36%	6%
EC, Turb, DO, Temp, Chl	0.0999	0.7864	0.0639	0.8265	36%	5%
EC, Turb, DO, Temp, Chl, pH	0.0907	0.8219	0.0581	0.8566	36%	4%
**TRP in River Enborne**						
EC	0.0666	0.5637	0.0396	0.7371	41%	24%
EC, DO	0.0608	0.6355	0.0258	0.8882	58%	28%
EC, DO, Temp	0.0343	0.8848	0.0178	0.9466	48%	7%
EC, DO, Temp, Turb	0.0257	0.9345	0.0161	0.9567	37%	2%
EC, DO, Temp, Turb, pH	0.0213	0.9559	0.0157	0.9587	26%	0%
EC, DO, Temp, Turb, pH, Chl	0.0212	0.9558	0.0162	0.9556	24%	0%
**NO_3_ in River Enborne**						
EC	0.0617	0.6107	0.0378	0.7113	39%	14%
EC, Temp	0.0559	0.6818	0.0206	0.9137	63%	25%
EC, Temp, pH	0.0274	0.9223	0.0138	0.9617	50%	4%
EC, Temp, pH, DO	0.0205	0.9566	0.0125	0.9684	39%	1%
EC, Temp, pH, DO, Turb	0.0172	0.9695	0.0119	0.9714	31%	0%
EC, Temp, pH, DO, Turb, Chl	0.0177	0.9681	0.0122	0.9704	31%	0%

## Data Availability

Publicly available datasets were analyzed in this study. The data can be found here: https://catalogue.ceh.ac.uk/documents/db695881-eabe-416c-b128-76691b2104d8, accessed on 12 December 2022.
